# Late Diagnosis of Silent Thoracic Aortic Rupture Presented as a Right Pleural Effusion

**DOI:** 10.1155/2012/906250

**Published:** 2012-12-20

**Authors:** Meletios A. Kanakis, Vassilios G. Papavassiliou, Polivios Drosos, Elias A. Kaperonis, George Benakis, Achilleas G. Lioulias

**Affiliations:** ^1^Thoracic Surgery Department, Sismanoglio General Hospital, 1 Sismanogliou Street, Marousi, 151 26 Athens, Greece; ^2^Department of Vascular Surgery, Sismanoglio General Hospital, 1 Sismanogliou Street, Marousi, 151 26 Athens, Greece

## Abstract

Patients with ruptured thoracic aortic aneurysm rarely present in a stable clinical condition. A man was referred to our hospital with the diagnosis of ruptured saccular aneurysm of the descending thoracic aorta. He successfully underwent both endovascular graft repair and open thoracotomy.

## 1. Introduction

Rupture of an aneurysm of the thoracic aorta is an emergency condition, which requires prompt diagnosis and treatment. However, in very rare cases, patients may present in stable clinical condition [1, 2]. We describe a patient with rupture of a descending thoracic aortic aneurysm into the right pleural cavity with some unusual clinical characteristics, making initial correct diagnosis more difficult. 

## 2. Case Report

**Figure 1:**
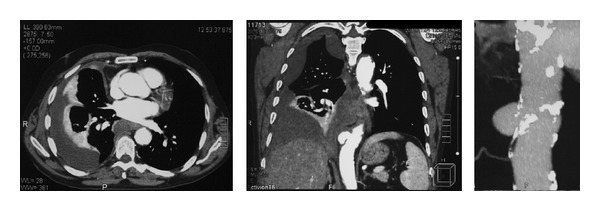
Computed tomographic scan of the chest depicting the right pleural effusion along with atelectasis and the saccular aneurysm of the descending aorta.

A 65-year-old male was admitted to a regional hospital complaining of progressive cough and respiratory discomfort. The patient had a disease-free history, but for a cervical abscess and mediastinitis due to neglected dental inflammation, for which he had been submitted to drainage through a right lateral cervical incision two years before. Chest radiography revealed right pleural effusion. Apart from anemia (Ht: 32%) and elevated CRP, all other parameters were normal. Symptoms recessed after a total of 1,000 mL nonclotting bloody fluid drainage through thoracentesis. The fluid analysis showed hematocrit 6.2%, WBC 2.4 × 10 cells/L with 22% neutrophils and 75% lymphocytes, glucose 84 mg/dL, LDH 832 IU/L, and protein 5.5 g/dL, and its cytologic examination was negative for malignancy. The differential diagnosis was quite broad, including most lesions producing exudative fluid such as tumors of the lung, pleura, or mediastinum. During the next days the patient was mildly febrile, while cough and respiratory discomfort regressed. Computed tomographic scan of the chest confirmed the right pleural effusion along with atelectasis and revealed a saccular aneurysm of the descending aorta in front of T9 vertebra (Figure 1). It measured 2.4 cm, 1.5 cm, and 2.6 cm in longitudinal, transverse, and oblique diameters, respectively, and communicated with thoracic aorta through a lumen of 16 × 10 mm width. The aortic diameter above the orifice was 32 mm and below the orifice 30 mm. The radiologist assumed that minor rupture might exist. The patient was transferred to a tertiary hospital immediately.

Upon arrival at our department, the patient experienced respiratory stress necessitating chest tube drainage of the right hemithorax a few hours later. At insertion of the chest tube, clots along with nonclotting bloody fluid were noted. Chest radiography showed right hemithorax opacity and atelectasis. The patient presented low evening fever, while blood culture was negative. A combined repair was decided. Under general anesthesia, first, a thoracic endovascular graft (GORETAG thoracic endoprosthesis) was introduced through the right common femoral artery into the descending thoracic aorta at the region of the saccular aneurysm under direct fluoroscopic guidance. The size of the endograft was based on these measurements plus an oversize of around 15% and enough length to secure safe landing and minimize the risk of endoleak. The device was the straight nontapered GoreTag thoracic endoprosthesis with 37 mm diameter and 10 cm length. After deployment of the endograft minimal endoleak was still visible. Ballooning with the trilobe balloon improved wall apposition of the stent graft. Completion angiography showed excellent patency of the endograft without any sign of endoleak. Right after, a right thoracotomy was performed. A large amount of clots were removed. At the region of the saccular aneurysm multiple postinflammatory mediastinal adhesions were recognized. The postoperative course was uneventful, and the patient was discharged from the hospital 8 days after the operation.

**Figure 2:**
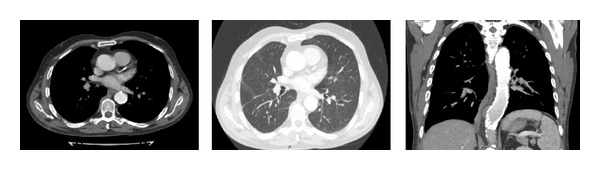
A follow-up CT angiography, three months later showed a smooth and patent endograft with no migration or endoleak. The lung is fully re-expanded.

A follow-up CT angiography, three months later, showed a smooth and patent endograft with no migration or endoleak (Figure 2). The patient has returned to normal life activity.

## 3. Discussion

Most atherosclerotic aneurysms are found in patients who are older than 60 years. They are asymptomatic and are found as a mediastinal mass during routine examination [3, 4]. Symptoms and signs are usually related to rapid enlargement, which creates pressure on surrounding tissues and structures. 

Aortic dissection or rupture is a major reported cause of hemothorax, which usually presented on left side for anatomical reasons [5]. There are some cases of right-sided hemothorax due to ruptured descending thoracic aneurysm of nontraumatic origin that have been described in the English literature [1, 2, 6, 7]. Most of them had an acute presentation and needed urgent surgical management. Baharloo et al. described a patient with a right-sided bleeding due to a fusiform aneurysm of the descending thoracic aorta ruptured along the right side of its wall, causing, however, hemorrhagic shock. The formation of a clot at the site of the tear prevented fatal exsanguinations [2]. In the case of the present patient, hemorrhagic shock was absent, and the patient remained undiagnosed for a period of 10 days. The mediastinal adhesions due to his previous mediastinitis possibly played a major role in hampering continuous bleeding from the ruptured aneurysmal wall.

Once the diagnosis of aortic rupture is established, a multidisciplinary decision for further management with surgery or endovascular stenting should be undertaken. Several factors need to be taken into account, including the patient's risk factors, patient's status (stable or unstable), and institutional factors (availability and resources). Open surgical repair is performed via a left posterolateral thoracotomy with or without cardiopulmonary bypass support and postoperative mortality ranges from 15% to 30%, and the incidence of paraplegia is reported to be 14% [8]. Endovascular stent grafting approach has at least equal short-term results in comparison with traditional open surgical repair and can be considered a safe alternative treatment modality in the therapeutic algorithm of thoracic aortic injury [8, 9]. The right-sided rupture of thoracic aorta was a significant factor for choosing the endovascular stent grafting approach. Furthermore, the right thoracotomy was imperative due to the long presence of clots in the thoracic cavity, which impaired lung expansion and would probably lead to empyema and subsequent trapped lung. 

The diagnosis of a ruptured thoracic aorta can be misdiagnosed due to its atypical clinical presentation. A thoracic aortic rupture should always be included in differential diagnosis even in a stable patient with right hemorrhagic pleural effusion. Furthermore, the current therapeutic armamentarium offers excellent results by endovascular management of thoracic aorta.
